# Chronic Kidney Disease Epidemic in Central America: Urgent Public Health Action Is Needed amid Causal Uncertainty

**DOI:** 10.1371/journal.pntd.0003019

**Published:** 2014-08-07

**Authors:** Pedro Ordunez, Ramón Martinez, Ludovic Reveiz, Evelina Chapman, Carla Saenz, Agnes Soares da Silva, Francisco Becerra

**Affiliations:** Pan American Health Organization, Washington, D.C., United States of America; University of California San Diego School of Medicine, United States of America

The 52nd Directing Council of the Pan American Health Organization (PAHO), in response to a call for action of the Minister of Health of El Salvador, recognized chronic kidney disease from nontraditional causes (CKDnT) affecting agricultural communities in Central America as a serious public health problem that requires urgent, effective, and concerted multisectoral action [1].

Most Central American countries do not have surveillance systems capable of detecting chronic kidney disease (CKD). However, many reports [2]–[4] and data from PAHO show the epidemiological magnitude of the disease. A proxy for CKDnT mortality, the age standardized mortality rate due to chronic kidney disease—coded as N18 (CKD-N18) by the 2010 International Classification of Diseases—is notably higher for men and women in Nicaragua and El Salvador compared to other countries in the region and has been since at least 2000 (http://www.paho.org/hq/index.php?option=com_content&view=article&id=9402). CKD-N18 data also show disproportionate mortality from the disease in males compared to females (Figure 1). Mortality due to CKD in El Salvador and Nicaragua exhibited a pattern of excess mortality in young adults (Figure 2), which is consistent with many other clinical and epidemiological reports [2]–[4].

**Figure 1:**
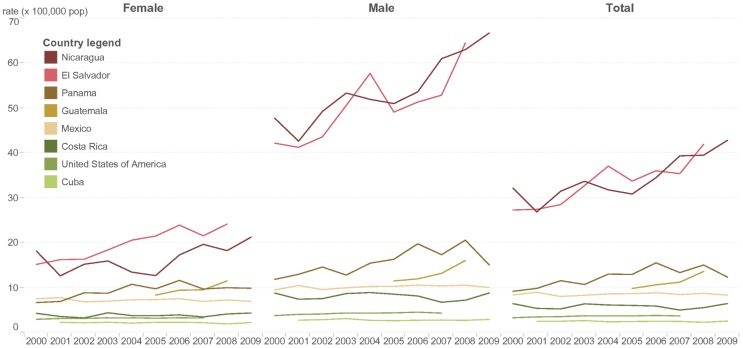
Chronic kidney disease (N18; International Classification of Diseases, tenth revision [ICD-10]) age-standardized mortality rate, selected countries, 2000–2009. Chronic kidney disease (N18, ICD-10) age-standardized mortality rates show a disproportionate mortality in males compared to females. Nicaragua and El Salvador have higher mortality rates, with an extreme excess in males compared to the rest of the countries. Source: Regional Mortality Database. PAHO, World Health Organization (WHO); 2014.

**Figure 2:**
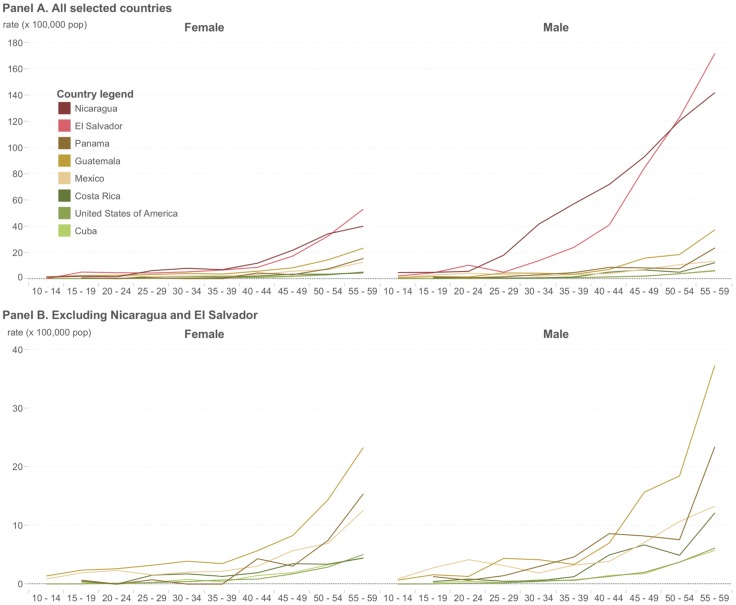
Chronic kidney disease (N18, ICD-10) age-specific mortality rate, selected countries, around 2008. (A) Mortality due to CKD (N18, ICD-10) in El Salvador and Nicaragua exhibited a pattern of excess mortality in young adults, starting at ages 25–29 years old. (B) Removing El Salvador and Nicaragua, panel B shows other countries of Central America also have an excess of premature mortality. Source: Regional Mortality Database. PAHO, WHO; 2014.

CKDnT has been largely reported in some clustered farming communities traditionally burdened by socioeconomic disadvantages from northern Nicaragua [2], the Pacific coast of El Salvador [3], and other countries such as Costa Rica, Guatemala, Honduras, and the south of Mexico [4]. The disease affects mostly young adult male agricultural workers, e.g., sugarcane cutters. CKDnT has also been described in agricultural workers in Sri Lanka and India [5]–[6].

Studies reveal that patients affected by CKDnT show a clinical and pathologic pattern of a tubule-interstitial disease [7]–[8], which seems to progress to end-stage renal disease in a relatively short time. This clinical pattern explains at least in part the high burden imposed on the affected countries for the delivery of health services. For example, a 50% increase in hospitalizations for CKD from 2005 to 2012 was reported in El Salvador, making CKD the leading cause of death in El Salvador's main hospital [1].

CKDnT is a chronic and multifactorial condition that has been neglected for quite some time. The causes of this epidemic have not been elucidated yet. Several potential etiological factors have been considered [7]. Given the disease's higher prevalence in agricultural communities and its clinical and epidemiological characteristics, which are similar to CKDnT in Sri Lanka [5], it is reasonable to draw attention to two interdependent factors: the misuse of agrochemicals and the working conditions of the labor force.

The misuse of pesticides has been widespread in Central America for a long time [9]. This region imported 33 million kg of active ingredient per year with an increase of 33% during 2000–2004. From a total of 403 pesticides (13 of which constitute 77% of the total pesticides that were imported), 22% were highly/extremely acutely toxic, 33% were moderately/severely irritating or sensitizing, and 30% had multiple chronic toxicities. Out of 41 banned or highly regulated pesticides as per international treaties, 16 were imported to Central America, four of which are among the 13 most imported pesticides [10]. Although the specific mechanisms to explain the nephrotoxicity of some pesticides are still under investigation, the nephrotoxicity of several of them is already known [11], [12].

Harsh working conditions, especially regular exposure to very hot temperatures and extreme physical effort, lead to heat stress and dehydration. Along with exposure to pesticides, these seem to play an important role in the occurrence of the disease, particularly among sugarcane cutters [13]. The weakness of regulatory systems [10], along with the agriculture dependency of local economies [14] and cultural agricultural practices [9], contribute to poor compliance with international safety and health standards for the use of agrochemicals and for occupational hygiene.

Many questions related to these potential causative agents remain unanswered. For example, why is there such an important difference in the distribution of CKD between countries? Are there differences in the agricultural practices and work processes in areas with the same climatic characteristics and devoted to the same type of plantations (e.g., sugarcane)? Which types of agrochemicals have been used in the affected areas? How do we explain cases in nonsugarcane cutters, as well as in women?

Other hypothesized causal agents merit further investigation. Nonsteroidal anti-inflammatory drugs, alcohol, and sugary beverage consumption have been associated with the disease [7], but their role remains controversial in current scientific evidence. The potential role of heavy metals and contamination of fertilizer has not been investigated in depth in the region and deserves more research. It has been argued that infectious diseases such as leptospirosis and dengue, which are prevalent in the region, could also play a role in the CKDnT epidemic [7]. However, these hypothesis have not been supported by evidence. Indeed, the human transmission of the West Nile virus, which has been associated with CKD [15], has not been documented in Central America until now.

A CKDnT regional research agenda is imperative not only to drive efforts to determine the epidemic's causative agents but also to bridge the gap between research and public health interventions. However, much-needed research must not delay action to address CKDnT. The resolution on CKDnT in Central America approved by PAHO's Directing Council [1] commits to coordinated and evidence-informed action to implement public policies, programs, and regulatory mechanisms to improve the social, environmental, occupational, and economic conditions of the affected communities and to strengthen surveillance and CKD-relevant health services.

The resolution of PAHO [1] also highlighted the relevance of multisectorial actions outside of the health sector—for instance, agriculture, trade, environment, occupational safety, affected communities, academia, and civil society, among others—to coordinate efforts, mobilize resources, prioritize the sustainability of actions to promote evidence-based public policies, and to reach the high level of commitment to reduce environmental risk factors to mitigate, on an urgent basis, the health, social, and economic consequences of this disease. An effective and urgent response to address and ultimately stop the epidemic is a moral duty not only for Central America but for the whole Pan American community.

## References

[1] Pan American Health Organization (2014) Resolution CD52.R1. Chronic kidney disease in agricultural communities in Central America. Washington, D.C. 2013 Available: http://www.paho.org/hq/index.php?option=com_content&view=article&id=8833&Itemid=40033&lang=en Accessed 21 March 2013..

[2] Torres C, Aragón A, González M, López I, Jakobsson K, et al (2010) Decreased Kidney Function of Unknown Cause in Nicaragua: A Community-Based Survey. Am J Kidney Dis 55: 485–496. 20116154 10.1053/j.ajkd.2009.12.012

[3] Orantes CM, Herrera R, Almaguer M, Brizuela EG, Hernández CE, et al (2011) Chronic kidney disease and associated risk factors in the Bajo Lempa region of El Salvador: Nefrolempa study, 2009. MEDICC Rev 13: 14–22. 10.37757/MR2011V13.N4.5 22143603

[4] Weiner DE, McClean MD, Kaufman JS, Brooks DR (2013) The Central American epidemic of CKD. Clin J Am Soc Nephrol 8: 504–511. 23099656 10.2215/CJN.05050512

[5] Jayatilake N, Mendis S, Maheepala P, Mehta FR, CKDu National Research Project Team (2013) Chronic kidney disease of uncertain aetiology: prevalence and causative factors in a developing country. BMC Nephrol 14: 180 10.1186/1471-2369-14-180 23981540 PMC3765913

[6] Reddy DV, Gunasekar A (2013) Chronic kidney disease in two coastal districts of Andhra Pradesh, India: Role of drinking water. Environ Geochem Health 35: 439–454 10.1007/s10653-012-9506-7 23475496

[7] Correa-Rotter R, Wesseling C, Johnson RJ (2014) CKD of Unknown Origin in Central America: The Case for a Mesoamerican Nephropathy. Am J Kidney Dis 63: 506–520. 24412050 10.1053/j.ajkd.2013.10.062 PMC7115712

[8] Wijkström J, Leiva R, Elinder CG, Leiva S, Trujillo Z, et al (2013) Clinical and Pathological Characterization of Mesoamerican Nephropathy: A New Kidney Disease in Central America. Am J Kidney Dis 62: 908–918. 23850447 10.1053/j.ajkd.2013.05.019

[9] Aragon A, Aragón C, Thörn Å (2001) Pests, peasants, and pesticides on the northern Nicaroguan Pacific plain. Int J Occup Environ Health 7: 295–302. 11783859 10.1179/107735201800339281

[10] Bravo V, Rodríguez T, van Wendel de Joode B, Canto N, Calderón GR, et al (2011) Monitoring Pesticide Use and Associated Health Hazards in Central America. Int J Occup Environ Health 17: 258–269. 21905395 10.1179/107735211799041896

[11] Li Q, Peng X, Yang H, Wang H, Shu Y (2011) Deficiency of Multidrug and Toxin Extrusion 1 Enhances Renal Accumulation of Paraquat and Deteriorates Kidney Injury in Mice. Mol Pharm 8: 2476–2483. 21991918 10.1021/mp200395f PMC3230245

[12] Siddharth M, Datta SK, Bansal S, Mustafa M, Banerjee BD, et al (2012) Study on organochlorine pesticide levels in chronic kidney disease patients: association with estimated glomerular filtration rate and oxidative stress. J Biochem Mol Toxicol 26: 241–247. 22645066 10.1002/jbt.21416

[13] Crowe J, Wesseling C, Solano BR, Umaña MP, Ramírez AR, et al (2013) Heat exposure in sugarcane harvesters in Costa Rica. Am J Ind Med 56: 1157–1164. 23775893 10.1002/ajim.22204

[14] Rosenthal E (2005) Who's afraid of national laws? Pesticide corporations use trade negotiations to avoid bans and undercut public health protections in Central America. Int J Occup Environ Health 11: 437–443. 16350478 10.1179/oeh.2005.11.4.437

[15] Nolan MS, Podoll AS, Hause AM, Akers KM, Finkel KW, et al (2012) Prevalence of Chronic Kidney Disease and Progression of Disease Over Time among Patients Enrolled in the Houston West Nile Virus Cohort. PLoS ONE 7: e40374 10.1371/journal.pone.0040374 22792293 PMC3391259

